# A Dual-Mode Wireless Microsystem for Monitoring Dopamine and Spike Changes with Dexmedetomidine

**DOI:** 10.34133/cbsystems.0566

**Published:** 2026-05-21

**Authors:** Peiyao Jiao, Yilin Song, Jin Shan, Yu Liu, Qianli Jia, Qi Li, Ying Wang, Yan Luo, Pengfei Zhao, Juntao Liu, Zhenchang Wang, Mixia Wang, Xinxia Cai

**Affiliations:** ^1^ State Key Laboratory of Transducer Technology, Aerospace Information Research Institute, Chinese Academy of Sciences, Beijing 100190, China.; ^2^ School of Electronic, Electrical and Communication Engineering, University of Chinese Academy of Sciences, Beijing 100049, China.; ^3^ Department of Anesthesiology, Ruijin Hospital, Shanghai Jiaotong University School of Medicine, Shanghai 200025, China.; ^4^ Department of Radiology, Beijing Friendship Hospital, Capital Medical University, Beijing 100050, China.

## Abstract

To understand how cortical circuits respond to pharmacological regulation, tools that can simultaneously detect electrophysiological and electrochemical signals in vivo are needed. However, most existing methods target only a single modality or depend on tethered recording systems that constrain movement and limit the ability to monitor coordinated neural processes. To address these challenges, we developed a dual-mode wireless microsystem that enables simultaneous recording of spikes, local field potentials (LFPs), and dopamine (DA)-related electrochemical signals on microelectrode arrays. The platform integrates a PtNPs/PEDOT:PSS/rGO/Nafion-modified electrochemical site for sensitive and selective detection of DA, as well as independent electrophysiological and electrochemical acquisition pathways that support stable long-distance wireless transmission of dual-mode signals. In vitro tests demonstrated that the platform can stably detect DA within a certain range and exhibits reliable wireless transmission performance. Using this platform, we recorded simultaneous electrophysiological and dopaminergic signals from the prelimbic cortex under different doses of dexmedetomidine. The results showed that increasing drug dose led to a significant reduction in spike firing rate and high-frequency LFP power, accompanied by dose-dependent elevations in DA-related amperometric response. These combined measurements showed simultaneous dose-dependent changes in electrophysiological activity and the DA-related electrochemical signal under dexmedetomidine. This dual-mode wireless microsystem provides a practical tool for neuroscience experiments requiring the integration of electrophysiology and neurochemistry.

## Introduction

Simultaneously acquiring electrophysiological (EP) and electrochemical (EC) signals is crucial for understanding how neural circuits respond to changes in internal states and pharmacological interventions [1–3]. Electrophysiological signals provide high-resolution information about neuronal firing (spikes) and local field potentials (LFPs), while electrochemical measurements are used to monitor dynamic fluctuations in neurotransmitters like dopamine (DA). Simultaneous recording of both modalities within the same cortical region allows researchers to jointly characterize rapid neuronal activity with relatively slower neurotransmitter changes, thus providing a more complete view of brain state transitions in vivo [4,5]. The medial prefrontal cortex (mPFC), particularly the prelimbic (PrL) area, is a key region involved in regulating arousal levels, anesthetic responses, and cognitive processing, making it an important target for dual-mode studies [6–8].

Although electrophysiological and electrochemical sensing technologies have made substantial progress in their respective fields, integrating both modalities into a compact wireless platform still presents considerable technical challenges. While wired systems can provide high-fidelity signals, they restrict animal movement, introduce mechanical artifacts, and are unsuitable for long-distance recording scenarios. Existing wireless microsystems primarily support only electrophysiological recording [9–13], with only a few platforms integrating electrochemical sensing capabilities [14,15]. These platforms often suffer from limitations such as restricted sampling rates, short transmission distances, or a lack of dual-modal simultaneous acquisition. Electrochemical DA detection has been achieved through various methods [16–18]; however, these methods often rely on benchtop workstations or optical instruments, thus failing to meet the needs of untethered in vivo experiments. As a result, few wireless microsystems can reliably achieve simultaneous acquisition of electrophysiological and neurotransmitter signals with stable performance over a practically meaningful transmission distance. This technological gap limits neuroscience research that requires highly coupled studies of electrophysiological and neurochemical signals.

Dexmedetomidine is a widely used sedative and analgesic drug, and its effects on cortical activity and neurotransmitter release remain a hot topic of current research [19,20]. Among many neurotransmitters, DA was selected because it is commonly examined in the context of dexmedetomidine-related responses [21,22] and is widely studied with established in vivo electrochemical methods [4,23,24]. Previous studies have shown that this drug can significantly inhibit neuronal firing and promote low-frequency LFP activity [25–27], but its influence on DA signaling shows inconsistent results across different measurement techniques. Microdialysis studies typically report that dexmedetomidine reduces extracellular DA levels [28,29], whereas recent works based on fiber photometry suggest that dopaminergic activity may be enhanced [21,30]. This discrepancy highlights the urgent need for minimally invasive, time-resolved, and multimodal simultaneous measurement tools. A wireless platform capable of simultaneously capturing electrophysiological and DA-related electrochemical changes would provide new insight into how dexmedetomidine modulates neuronal firing and neurotransmitter dynamics within PrL.

**Fig. 1.**
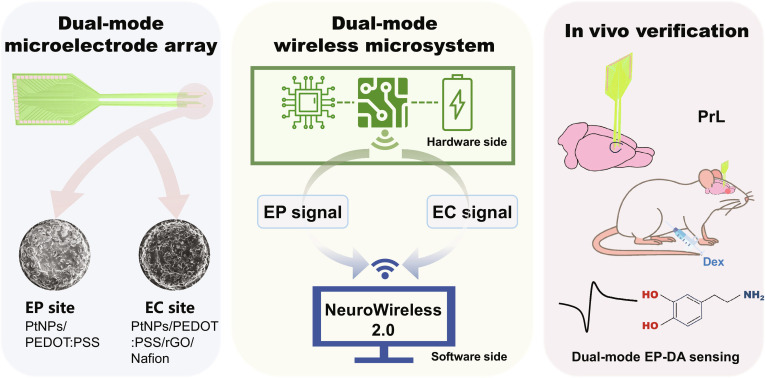
Three main modules of this work. 1. Dual-mode microelectrode array: incorporates electrophysiological (EP) sites modified with PtNPs/PEDOT:PSS and an electrochemical (EC) site for dopamine (DA) sensing modified with PtNPs/PEDOT:PSS/rGO/Nafion. 2. Dual-mode wireless microsystem: features dual-mode signal acquisition using EP and EC front-ends, dual-channel wireless transmission based on FPGA-ESP32 architecture, and coordinated data handling through the NeuroWireless 2.0 platform. 3. In vivo verification: enables simultaneous recording of spikes, LFPs, and DA-related electrochemical signals in the prelimbic (PrL) cortex during dexmedetomidine (Dex) administration. The 3 subsystems form a closed pipeline enabling end-to-end dual-mode neural monitoring.

Here, we present a dual-mode wireless microsystem that enables simultaneous recording of electrophysiological and electrochemical signals in vivo (Fig. 1). Our work integrates a dual-mode microelectrode array (MEA) for spikes, LFPs, and DA detection; a compact wireless microsystem featuring separate electrophysiology and electrochemical acquisition paths; and a long-range wireless transmission module for stable data streaming. A platinum nanoparticles (PtNPs)/poly (3,4-ethylenedioxythiophene) polystyrene sulfonate (PEDOT:PSS)/ reduced graphene oxide (rGO)/Nafion-modified electrochemical site provides sensitive DA detection, and the dual-controller architecture supports concurrent high-throughput electrophysiology and low-bandwidth electrochemical acquisition. Using this platform, we demonstrate in vitro wireless DA calibration and in vivo dual-mode recording from PrL across graded dexmedetomidine administration. These results demonstrate the good practicality and versatility of this method in integrated monitoring of neural signals, providing a basis for studying cortical state changes involving neurotransmitters.

## Methods

### Modification of the electrochemical site on MEA

The MEA used in this study, including its parameters, was identical to that reported previously, and its design and fabrication procedures can be found in Ref. [31]. The electrophysiology sites were modified following the same protocol in Ref. [31], using sequential electrodeposition of PtNPs and PEDOT:PSS, which has been shown to provide good biocompatibility and a stable signal-to-noise ratio in in vivo recordings [32].

For the electrochemical sites, an rGO layer was added on the PtNPs/PEDOT:PSS to improve sensitivity for DA detection. The stability of PtNPs/PEDOT:PSS and PtNPs/rGO composite modifications has been evaluated in our previous studies over 14 d [33] and 10 d [24], respectively. rGO was mixed with PEDOT:PSS and ultrasonicated to form a uniform electroplating solution. The sites were modified using a 3-electrode setup, with the electrochemical site as the working electrode (WE), a platinum wire as the counter electrode (CE), and an Ag/AgCl reference electrode (RE). Cyclic voltammetry (CV) was performed from 0 to 0.9 V at a scan rate of 100 mV/s for 5 cycles to electrodeposit the composite layer [30]. After electrodeposition, 5 μl of Nafion solution (prepared in 20 μl anhydrous ethanol) was drop-casted onto the microelectrode surface to reduce interference from other electroactive species during in vivo measurements [23,34].

### Architecture of the dual-mode wireless microsystem

**Fig. 2.**
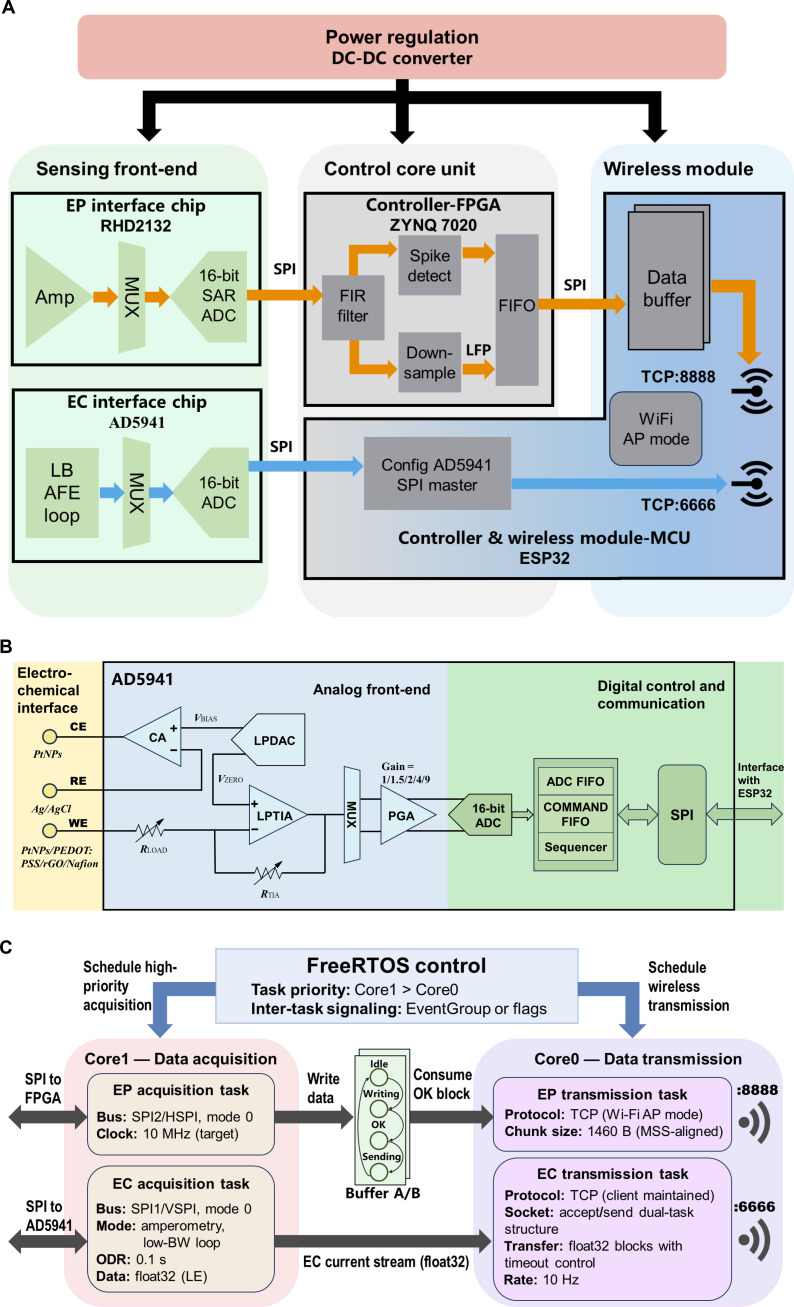
Architecture and wireless operation of the dual-mode microsystem. (A) Block diagram of the microsystem integrating electrophysiological (EP) and electrochemical (EC) acquisition pathways with FPGA and ESP32 controllers. (B) Functional diagram of the AD5941 amperometric front-end used for dopamine detection. (C) ESP32 task scheduling and dual-TCP wireless data-streaming scheme for simultaneous EP and EC transmission.

The architecture of the microsystem is shown in Fig. 2A. The overall microsystem design followed the framework reported in our previous work [31], which integrated an RHD2132-based electrophysiology interface chip, a field programmable gate array (FPGA) core unit, an ESP32 wireless module, and a power regulation module. In this study, the microsystem was extended by introducing AD5941 as an electrochemical acquisition front-end, enabling the microsystem to support simultaneous dual-mode signal recording [18]. AD5941 was configured directly through the ESP32 microcontroller unit (MCU), which also read the 16-bit electrochemical data through an additional serial peripheral interface (SPI). FPGA and ESP32 served as 2 independent control units within the microsystem. Electrophysiological and electrochemical data were transmitted over separate transmission control protocol (TCP) ports, ensuring that the 2 data streams did not interfere with each other.

### Wireless electrochemical detection methods

As shown in Fig. 2B, electrochemical DA detection was performed using AD5941 in amperometric mode. On the electrochemical interface side, the CE and WE terminals of AD5941 were connected to 2 sites on MEA modified with PtNPs and PtNPs/PEDOT:PSS/rGO, respectively, while the RE terminal was connected to an external Ag/AgCl wire. In the analog front-end (AFE) of AD5941, the low-power digital-to-analog converter (LPDAC) generated 2 voltages, Vbias and Vzero, whose difference was configured to match the oxidation potential of DA. This constant potential was applied between RE and WE. The oxidation current at WE was converted to a voltage by the low-power transimpedance amplifier (LPTIA) and subsequently amplified by the programmable gain amplifier (PGA). The conditioned signal was converted to digital form by the integrated 16-bit analog-to-digital converter (ADC).

As shown in Fig. 2C, AD5941 was controlled directly by ESP32 via a dedicated SPI. At system startup, ESP32 initialized AFE according to the amperometric mode requirements. During acquisition, ESP32 polled AD5941 at a fixed interval and read each data through its SPI. The AD5941 application firmware converted the raw ADC codes (16 bits) into current values (μA) in single-precision floating-point (float32) format and stored them in an on-chip buffer. These 32-bit floating-point samples were then transmitted from ESP32 to personal computer (PC) via TCP (port 6666).

The ESP32’s dual-core architecture enabled deterministic task scheduling. Core 1 was dedicated to data acquisition (high priority), running 2 independent tasks responsible for electrophysiological and electrochemical signal acquisition, respectively. Core 0 handled wireless transmission and used 2 TCP transmission tasks serving ports 8888 (EP) and 6666 (EC). FreeRTOS was used for inter-task signaling, ensuring that electrochemical data are only sent after they have been successfully acquired from AD5941. Because electrochemical sampling occurred at a low rate (10 Hz), EC data were sent immediately without double-buffering, whereas electrophysiology relied on a ping-pong buffer mechanism for high-throughput streaming. This separation of buses, cores, and TCP ports prevented modality interference and enabled stable dual-mode operation.

On the software side, we extended our previous NeuroWireless platform to develop NeuroWireless 2.0, which added support for the additional electrochemical TCP stream. The software continuously listened to both ports, additionally received float32 electrochemical signals, displayed, and was able to store them for offline analysis. The user interface for EC is shown in Fig. S1.

### In vivo experimental procedures

A total of 5 male Sprague–Dawley rats (250 to 300 g) were used in this study. Animals were housed individually in individually ventilated cages (IVCs) (42 cm × 28 cm × 27 cm) under a 12-h light/12-h dark cycle with free access to food and water.

Following the standard animal surgical procedures, the surgery proceeded through the following steps. Each rat was first anesthetized with 5% isoflurane for induction and maintained at 0.6% to 1% during the operation. After a midline scalp incision and exposure of the skull, a craniotomy (approximately 3 mm × 3 mm) was made above the target region [anterior-posterior (AP): 2.8 mm; medial-lateral (ML): 0.8 mm; right hemisphere]. Four skull screws were placed around the craniotomy to provide mechanical support and grounding. An Ag/AgCl wire serving as the RE was inserted posterior to the lambda. MEA was then slowly lowered into PrL [dorso-ventral (DV) 3.0 mm from the dural surface]. Dental cement was applied to stabilize the array and secure all components. After the cement had fully hardened, an anti-inflammatory agent was administered intraperitoneally, and the animal was returned to its home cage for recovery for 1 to 2 d.

All experiments were carried out with the permission of the Beijing Association on Laboratory Animal Care and approved by the Institutional Animal Care and Use Committee at Aerospace Information Research Institute, Chinese Academy of Sciences (AIRCAS).

In each session, a baseline period of dual-mode signals was recorded first, followed by intraperitoneal injection of saline or dexmedetomidine (10, 40, or 100 μg/kg). Only one dose was administered per day, and each rat (*n* = 5) underwent 4 sessions on separate days (saline and 3 dexmedetomidine doses), thereby serving as its own control across conditions. Signal recording continued for an extended period after the injection. All injections were performed at approximately the same time of day to minimize the influence of circadian rhythms.

### Signal processing

Electrophysiological signals were preprocessed as described in our previous work [31]. Briefly, the electrophysiological signals were split into 2 paths by filtering. The raw recordings were high-pass filtered at 250 Hz, and spikes were detected using a threshold-based method; spike sorting was then performed offline using Offline Sorter (Plexon, USA) with the valley-seeking algorithm. In parallel, the raw recordings were low-pass filtered at 250 Hz to obtain LFPs. For LFP band analysis, delta was defined as 1 to 4 Hz, and gamma was defined as 30 to 60 Hz. Because the baseline DA-related amperometric current varied across sessions, we did not compare absolute current values between sessions. For each session, we took the mean current in a stable pre-injection window (I_pre) and in a stable post-injection window (I_post), and we report the normalized change as (I_post − I_pre)/I_pre. This within-session normalization reduces the impact of baseline offsets and slow drift on the group comparison.

### Statistical analysis

All data are presented as mean ± standard error (SE). Linear regression analysis was used to obtain the calibration curves of current versus DA concentration and to calculate the sensitivity and coefficient of determination (*R*^2^). For the in vivo dose comparisons (saline and 10, 40, and 100 μg/kg dexmedetomidine), the same animals were measured across conditions (one dose per day; *n* = 5 rats). Therefore, a one-way repeated-measures analysis of variance (ANOVA) was used for comparisons among doses, followed by post hoc multiple-comparison tests with correction for multiple testing. For spike firing rate, spike peak-to-peak amplitude, band-limited LFP power, and relative changes in the DA-related amperometric response, data were first summarized to one value per animal for each condition before statistical testing. Statistical analyses were carried out in Origin 2022 (OriginLab Corporation). A value of *P* < 0.05 was considered statistically significant. Significance levels are indicated in the figures as *P < 0.05, **P < 0.01, ***P < 0.001, and ns for no significant difference.

## Results

### Electrochemical and material characterization

To examine the interaction of DA with the modified materials, a series of in vitro electrochemical measurements was performed.

**Fig. 3.**
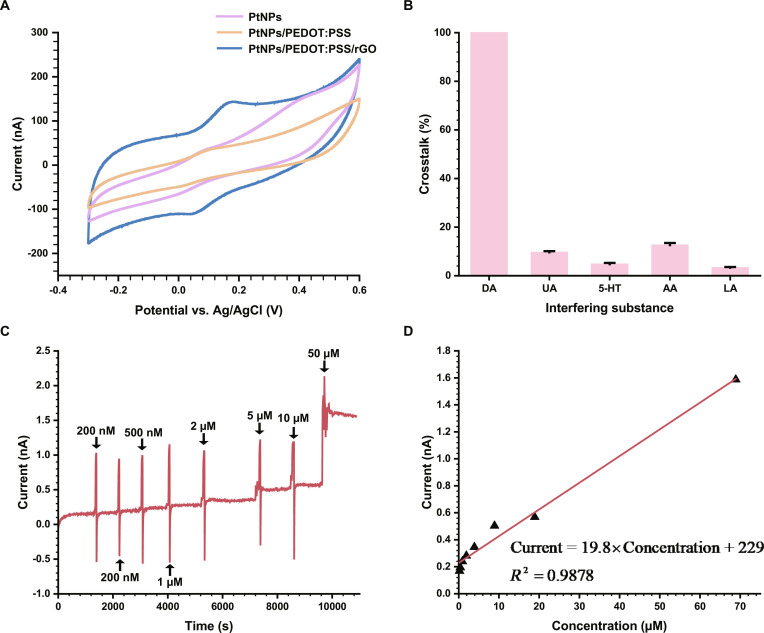
Electrochemical and material characterization of the dopamine (DA)-sensing site. (A) Cyclic voltammetry (CV) responses of microelectrodes with different modification schemes in 200 μM DA. (B) Comparison of the current response of DA in the presence of the corresponding interferent. (C) Wireless amperometric DA calibration measured through the microsystem. (D) Linear relationship between current and DA concentration based on data from (C).

Fig. 3A compares the CV responses of microelectrodes modified with PtNPs, PtNPs/PEDOT:PSS, and PtNPs/PEDOT:PSS/rGO in 200 μM DA solution. The rGO-composited microelectrode showed a more pronounced oxidation peak and a substantially larger oxidation current, and was therefore selected as the electrochemical site. Supporting material characterizations, including scanning electron microscope (SEM) morphology and energy-dispersive spectrometer (EDS) elemental analysis of different modification schemes, are provided in Fig. S2. Fig. S3 shows the CV curves of the PtNPs/PEDOT:PSS/rGO/Nafion-modified microelectrode recorded in phosphate-buffered saline (PBS) and 200 μM DA. The DA solution produced a pair of clear redox peaks, with the oxidation peak appearing at approximately 0.15 V versus Ag/AgCl. This potential was later applied between RE and WE in amperometric measurements to maximize DA oxidation while minimizing interference from other species.

The relationship between DA concentration and the measured current was further quantified using amperometric calibration. Fig. S4A shows the current responses to stepwise additions of DA, in which the incremental increase in concentration ranged from 10 nM to 10 μM. Fig. S4B presents a magnified view of the sensor responses in the low concentration range (10 to 200 nM), indicating that the detection limit of the PtNPs/PEDOT:PSS/rGO/Nafion-modified microelectrode was 10 nM. Based on the response currents measured from successive DA additions in Fig. S4A, a linear fit was obtained, as shown in Fig. S4C. The sensor exhibited a sensitivity of 32.4 pA/μM with a correlation coefficient of *R*^2^ = 0.99036, demonstrating stable performance and a clear concentration-dependent response.

To assess resistance to common interfering neurochemicals, uric acid (UA), 5-hydroxytryptamine (5-HT), ascorbic acid (AA), and lactic acid (LA) were individually added at the same concentration as DA. Their corresponding current responses are shown in Fig. S4D. DA generated a notable current response, while the other tested substances produced only minimal changes. As summarized in Fig. 3B, the DA response was markedly higher than that of the interferents, confirming that the PtNPs/PEDOT:PSS/rGO/Nafion-modified microelectrode provides excellent selectivity for DA detection. The high selectivity likely arises from the synergistic effect of the rGO mixed-conductivity network and the cation-exchange property of Nafion, which collectively promote DA oxidation while suppressing the access of anionic interferents.

Taken together, these results demonstrate that the modified electrochemical site exhibits high sensitivity, low detection limit, and strong selectivity, making it suitable for in vivo DA monitoring.

### Wireless performance of the dual-mode microsystem

#### Wireless amperometric DA calibration

The electrochemical sensing capability of the microsystem was evaluated through wireless calibration of DA, as shown in Fig. 3C. A series of DA solutions with increasing concentrations was introduced, and the corresponding oxidation currents were recorded through the wireless link. The resulting calibration curve is presented in Fig. 3D. A linear relationship was obtained between current and DA concentration, with a sensitivity of 19.8 pA/μM and a correlation coefficient of *R*^2^ = 0.9878. These results indicate that the wireless electrochemical pathway provides reliable quantitative detection performance, supporting its use for in vivo DA monitoring.

#### Hardware extension for dual-mode detection

**Fig. 4.**
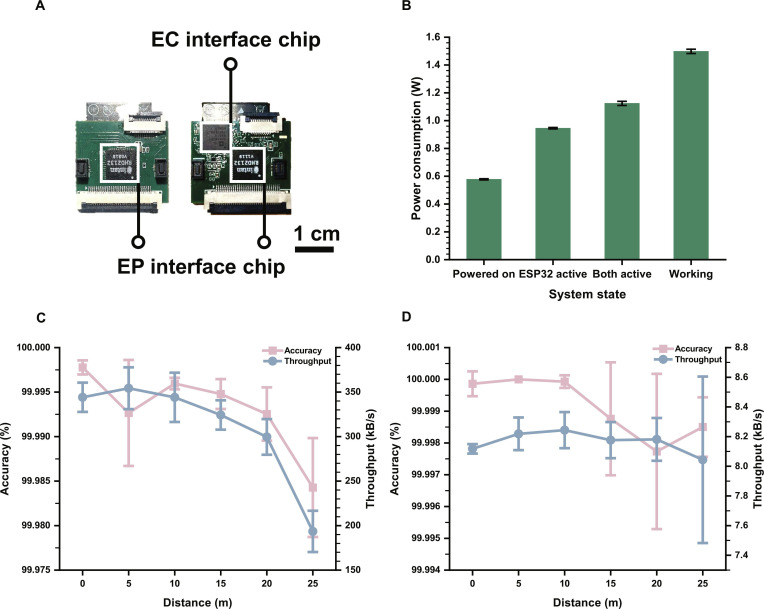
Wireless performance and operational characteristics of the dual-mode microsystem. (A) Photograph comparison of the updated PCB. The integration of the electrochemical (EC) interface chip increased board layers from 4 to 6 and slightly expanded the size (2.6 cm × 2.2 cm). EP, electrophysiological. (B) Power consumption in 4 operational states. (C) Wireless transmission accuracy and throughput of the EP pathway (TCP 8888) over distances of 0 to 25 m. (D) Wireless accuracy and throughput of the EC pathway (TCP 6666) over distances of 0 to 25 m.

The dual-mode wireless microsystem retained the stacked structure consisting of 2 printed circuit boards (PCBs), with board-to-board (BTB) connectors and a flexible printed circuit (FPC) outlet connected to MEA. The main hardware modification in this work was the addition of an EC interface chip to enable electrochemical sensing. As shown in Fig. 4A, the EC interface chip was integrated on the same side of the same PCB together with the existing EP interface chip. To accommodate the additional routing required by the EC pathway, the PCB stack-up was expanded from 4 to 6 layers. This change led to a small increase in board size from 2.4 cm × 2.2 cm to 2.6 cm × 2.2 cm, while overall mass increased from 8.68 g to 8.99 g.

#### Power consumption of the microsystem

The power consumption of the dual-mode microsystem was evaluated using a 3.7-V lithium battery as the power source. Power draw was measured under 4 operational states: Powered on, ESP32 active only, ESP32 and FPGA active (Both active), and Working. As shown in Fig. 4B, the power increased as more system components were engaged, reaching approximately 1.49 W in the Working state when both electrophysiological and electrochemical data were being processed and transmitted. This value is slightly higher than that observed in our previous single-mode microsystem, primarily due to the addition of a second TCP transmission pathway for electrochemical data. Based on this peak current, the microsystem offers about 37 min of working duration when powered by a 250-mAh lithium battery, matching the needs of most in vivo experiments.

#### Wireless transmission accuracy and throughput

Although our previous work evaluated the wireless transmission accuracy and throughput of a single-mode microsystem, the present study extends the architecture to dual SPI and dual TCP. It was therefore necessary to reassess wireless performance across different distances to ensure that both electrophysiological and electrochemical data can be transmitted reliably in practical animal-experiment environments. We adopted the same end-to-end testing approach as before, in which data were transmitted from FPGA to ESP32 and then wirelessly to PC.

To generate comprehensive test patterns, FPGA stored an 8-bit random sequence ranging from 0 to 255, covering all possible byte-level values in SPI transfers. The data were sent simultaneously through 2 SPIs to ESP32, corresponding to HSPI (representing the EP pathway) and VSPI (used to emulate communication between AD5941 and ESP32). Due to the different data-rate requirements of the 2, accuracy testing for the EP pathway (TCP port 8888) was performed using 1 MB of transmitted data, whereas the EC pathway (TCP port 6666) was evaluated with 1 kB of data. Wireless accuracy and throughput were measured at distances from 0 to 25 m in 5-m increments using the NeuroWireless 2.0 software platform.

The performance of ports 8888 and 6666 is shown in Fig. 4C and D, respectively. Both pathways maintained excellent transmission accuracy over the entire 0- to 25-m range, with the EP pathway achieving >99.98% accuracy and the EC pathway exceeding 99.998% accuracy. Based on the estimated throughput requirement from our previous work, the EP pathway needs a throughput of approximately 221 kB/s to support simultaneous LFP and spike streaming. The present microsystem meets this requirement at distances up to 20 m. For the EC pathway, a sampling rate of ~10 Hz is typically sufficient for amperometric DA detection, corresponding to a minimal required throughput of 40 B/s, which is easily satisfied by the measured performance.

Together, these results indicate that the dual-mode microsystem maintains reliable, high-quality wireless data transfer with adequate throughput over distances of at least 20 m, meeting the practical needs of in vivo neuroscience experiments.

#### Time alignment test between EP and EC data streams

To quantify the time alignment capability of the proposed dual-mode wireless streams, we measured the relative timing between the EP stream and the EC stream during simultaneous transmission. During the test, FPGA sent the same 100-byte payload with a shared sequence index to ESP32 through 2 SPIs, and ESP32 forwarded the 2 streams to PC through 2 TCPs. PC listened to the 2 TCP ports and recorded the arrival timestamp of every packet. Packets were then paired using the same sequence index, and the time difference between the paired EP and EC packets was calculated for each index. From this series of time differences, we computed the mean time offset (average delay difference), the jitter (standard deviation), and the drift trend by comparing the mean offset in the latter half of the test versus the former half.

With this procedure, the mean offset between the 2 streams was 0.717 ms, the jitter (standard deviation) was 1.42 ms, and the drift was −0.0734 ms. These results indicate that the EP and EC data can be aligned within a few milliseconds during wireless transmission, which is sufficient for the dual-mode demonstrations and descriptive analyses in this work.

#### Baseline drift test in PBS

We wirelessly recorded the amperometric current in PBS continuously for 50 min to evaluate baseline drift. As shown in Fig. S5, a slow baseline change was observed over time. The fitted drift rate was 0.126 pA/min. After removing the linear trend, the detrended root mean square (RMS) was 2.60 pA. This result shows that slow baseline drift exists during long recordings in PBS and should be considered in electrochemical data processing.

#### Cross-talk evaluation between EP and EC

We evaluated bidirectional cross-talk by enabling one modality while monitoring the other. As shown in Fig. S6A, in the EP→EC test, a baseline shift was observed in the PBS amperometric current after EP streaming was enabled. Using 2 time segments for quantification, the mean current changed from 45.6 pA (1 to 40 s) to 44.1 pA (100 to 140 s).

As shown in Fig. S6B, in the EC→EP test, no obvious increase of EP noise was observed after EC amperometric operation was enabled. Using 10-s windows, the EP demeaned RMS was (2.31 ± 0.0557) μV before EC and (2.26 ± 0.0653) μV after EC. These results indicate that, under the tested conditions, EC operation did not measurably increase EP noise, while EP operation can slightly shift the EC baseline.

### In vivo dual-mode electrophysiology-DA monitoring

**Fig. 5.**
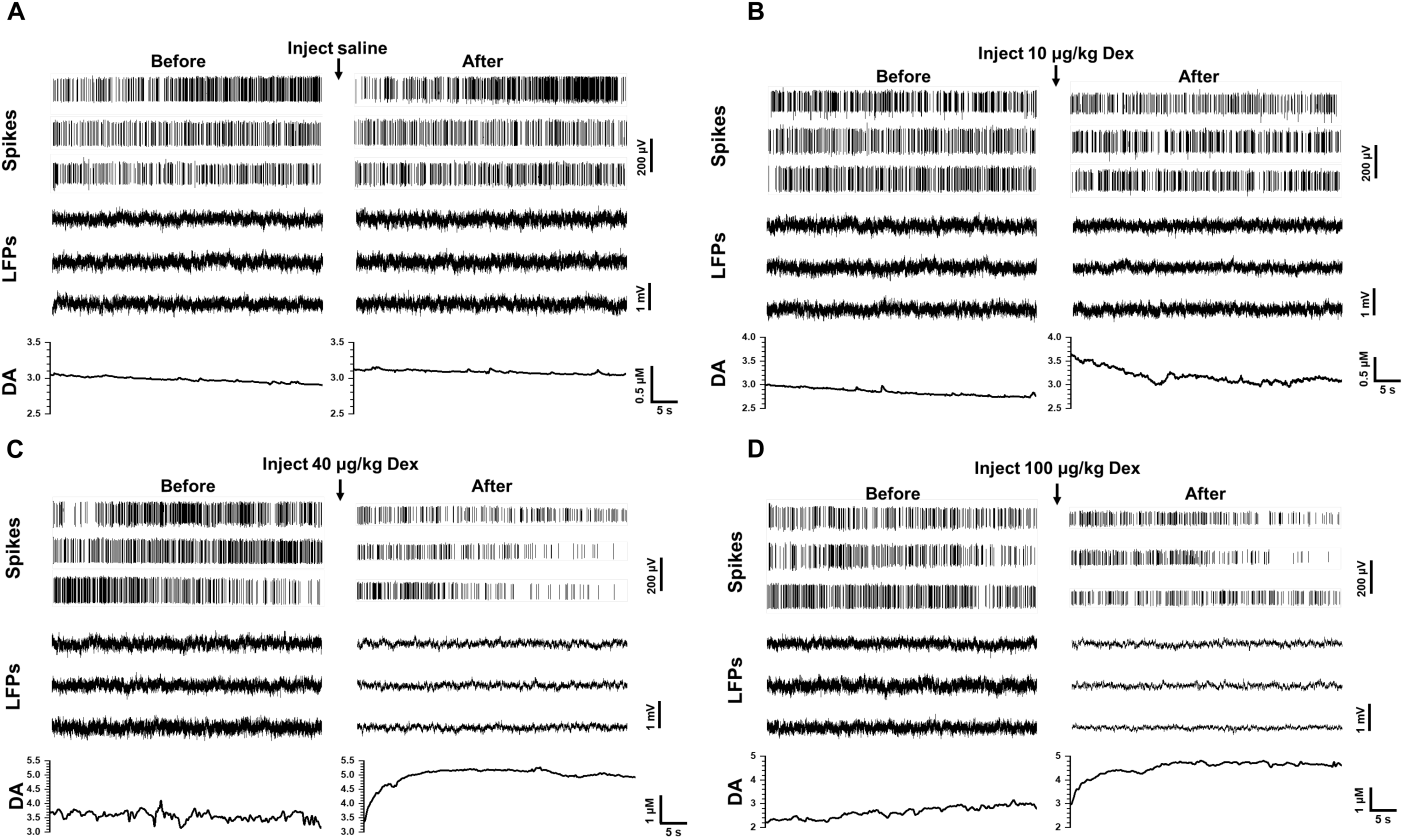
In vivo dual-mode electrophysiological and dopamine (DA) recordings before and after dexmedetomidine administration. (A to D) Representative simultaneously recorded spikes, local field potentials (LFPs), and DA-related amperometric response in the prelimbic cortex (PrL) before and after intraperitoneal injection of saline (A) and 10 μg/kg (B), 40 μg/kg (C), and 100 μg/kg (D) of dexmedetomidine. The DA values are converted to concentration using the in vitro calibration curve. These recordings demonstrate the microsystem’s ability to capture electrophysiological suppression and increased DA-related amperometric response.

Using the dual-mode MEA together with the dual-mode wireless microsystem, we recorded simultaneous electrophysiological and dopaminergic activity in PrL before and after intraperitoneal administration of dexmedetomidine. Fig. 5A to D shows representative multi-channel spike activity, LFP traces, and DA-related amperometric response obtained from rats receiving saline and 10, 40, and 100 μg/kg dexmedetomidine, respectively. These traces illustrate representative examples rather than population-averaged statistics. Across all channels, saline injection produced minimal changes in either electrophysiological activity or DA levels. In contrast, progressive dose-dependent effects were observed in the dexmedetomidine groups. Neural activities became increasingly suppressed, while DA-related amperometric response showed a clear upward trend in the 40 and 100 μg/kg groups. The next section provides quantitative analyses of these dual-mode changes.

These results demonstrate that the dual-mode wireless microsystem, combined with the dual-mode MEA, enables in vivo real-time and localized monitoring of electrophysiological signals and DA-related amperometric response.

### Analysis of dual-mode signal changes under dexmedetomidine administration

**Fig. 6.**
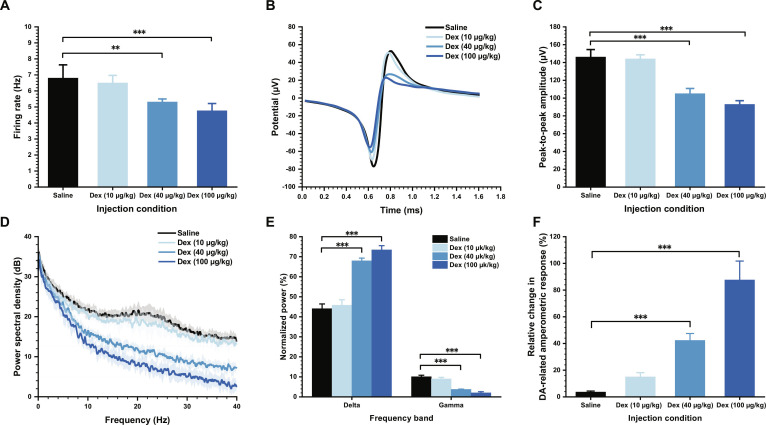
Quantitative analysis of dose-dependent electrophysiological and dopaminergic alterations under dexmedetomidine (Dex) administration. (A) Spike firing rate before and after dexmedetomidine injection at different doses. (B) Average spike waveforms across groups. (C) Peak-to-peak spike amplitudes before and after dexmedetomidine injection at different doses. (D) Local field potential (LFP) power spectral density (PSD) across groups. (E) Normalized delta- and gamma-band power of the LFPs. (F) Relative changes in dopamine (DA)-related amperometric response across groups with different doses.

We quantitatively analyzed the dose-dependent changes in the dual-mode signals before and after dexmedetomidine injection. The first and most sensitive indicator was the neuronal firing rate. As shown in Fig. 6A, spike firing progressively decreased with increasing dexmedetomidine dose, dropping from (6.80 ± 0.82) Hz in the control group to (6.50 ± 0.47) Hz at 10 μg/kg, (5.31 ± 0.18) Hz at 40 μg/kg, and (4.77 ± 0.45) Hz at 100 μg/kg. This gradual downward trend indicates that the sedative effect of PrL becomes more pronounced with increasing dosage.

We next examined the characteristics of single-unit spike waveforms. Fig. 6B shows the average spike waveform in each group, revealing a clear decrease in peak-to-peak amplitude with higher dexmedetomidine doses. Quantitative results are presented in Fig. 6C. The peak-to-peak amplitude remained stable in the control group [(146.13 ± 10.89) μV], showed a slight reduction at 10 μg/kg [(144.14 ± 9.03) μV], but dropped markedly at the standard dose [(104.95 ± 8.97) μV], corresponding to a decrease of approximately 28.2%.

LFP, reflecting population-level neural dynamics, also showed dose-dependent suppression. As shown in Fig. 5, the overall amplitude of the LFP decreased simultaneously with the weakening of single-unit firing activity. We further analyzed the spectral composition of LFPs, and the results are shown in Fig. 6D. The power spectral density (PSD) at different drug doses showed that the overall spectral power decreased with increasing sedation. Because the delta and gamma bands are particularly informative during sedative states, we performed a quantitative analysis of their normalized power. As shown in Fig. 6E, the proportion of the delta band increased significantly after administration of the 40 and 100 μg/kg doses, consistent with a transition toward slow-wave, low-arousal network activity. Meanwhile, the proportion of the gamma band decreased accordingly.

We analyzed the dynamic changes in DA-related amperometric response obtained through electrochemical recording. As shown in Fig. 6F, DA-related amperometric response exhibited a dose-dependent elevation, rising from 3.64% ± 0.74% in the control group to 15.01% ± 3.14% at 10 μg/kg and 42.31% ± 5.16% at 40 μg/kg, and reaching 87.63% ± 14.10% at 100 μg/kg. These results indicate that dexmedetomidine is associated with a dose-dependent increase in the DA-related amperometric response in PrL.

## Discussion and Conclusion

We developed a dual-mode wireless microsystem that enables concurrent in vivo acquisition of cortical electrophysiological signals (spikes and LFPs) and a DA-related amperometric response. The microsystem integrates an AD5941-based amperometric front end with independent wireless data pathways and supports stable streaming during in vivo recordings under graded dexmedetomidine dosing.

To place this work in context, the proposed wireless microsystem builds on our previous research, which focused solely on 32-channel electrophysiological recording, by adding an electrochemical sensing front-end and dual-TCP data pathways while preserving a compact, battery-powered commercial off-the-shelf (COTS)-based architecture. Representative related platforms and key system metrics are summarized in Table S1. Compared with typical wireless systems that either record only electrophysiology signals or combine electrophysiology with optical stimulation with a trade-off in terms of sampling rate or channel count, the present design maintains 32 channels at 30 kHz for spikes and LFPs and simultaneously supports continuous DA monitoring over a wireless solution that remains accurate and stable up to 20 m. In contrast to earlier electrochemical probes that rely on tethered potentiostats, our microsystem integrates the PtNPs/PEDOT:PSS/rGO/Nafion-modified site, on-board AD5941 front-end, and NeuroWireless 2.0 software into a single workflow, enabling end-to-end dual-mode acquisition, visualization, and storage without additional hardware. These features highlight the practical engineering advantages of this microsystem for integrated electrophysiological and electrochemical research.

Using this platform, we observed clear dose-dependent changes in electrophysiological and dopaminergic responses in PrL under dexmedetomidine administration. Neuronal firing rate and spike amplitude gradually decreased, LFPs shifted toward lower frequency components, and DA-related amperometric response increased through the same dose gradient. These patterns are consistent with current circuit-level understanding of dexmedetomidine. As a selective α2A-adrenergic receptor agonist, dexmedetomidine reduces locus coeruleus (LC) norepinephrine (NE) output [35,36], which lowers cortical excitability and promotes slow, synchronized network activity. At the same time, reduced noradrenergic input to the ventral tegmental area (VTA) can relieve inhibition on DA neurons, which may be associated with dopaminergic input to prefrontal regions. We observed a dose-dependent change in the DA-related electrochemical signal under dexmedetomidine, but this result should not be directly compared with earlier microdialysis reports because the 2 methods measure DA-related changes in very different ways [35]. Recent fiber-photometry studies have also reported dexmedetomidine-related changes in dopaminergic activity [21], which suggests that the reported direction and size of DA-related effects can vary across methods and experimental conditions. The present dual-mode wireless recordings provide simultaneous dual-mode observations under the same conditions. Notably, dexmedetomidine can also change systemic physiology (e.g., hemodynamics and respiration), which may affect both electrophysiological activity and the electrochemical readout; these systemic variables were not directly monitored or controlled in this study. Together, these results illustrate how neuromodulatory and circuit-level processes evolve in parallel across the sedation progression induced by dexmedetomidine.

However, several limitations remain in this study, and future work will aim to address them. The cohort size was limited (*n* = 5), which may reduce statistical power and does not fully capture inter-animal variability; therefore, the dose-related trends should be interpreted with appropriate caution. In addition, from the electrochemical measurement side, the in vivo readout should be interpreted carefully. In this work, a constant-potential amperometry (0.15 V) was used with an external Ag/AgCl reference. In vivo currents can be affected by factors beyond the target analyte, including electrode fouling and slow drift, local pH and oxygen changes, temperature fluctuations, fluid motion and injection-related transients, tissue reactions around the electrode, and possible reference instability. Although in vitro selectivity tests were performed and the analysis used within-session baseline normalization to reduce baseline offsets and slow drift, these steps cannot fully rule out in vivo confounds. For this reason, the neurochemical results are presented as a DA-related amperometric response rather than absolute extracellular DA concentration or DA release. In addition, independent validation with an orthogonal method (e.g., microdialysis, fast-scan cyclic voltammetry (FSCV), photometry, or post-mortem verification) and long-term tissue-response assessment were not included here and remain important directions for future work.

Beyond these limitations, several platform-level limitations also remain. First, although we optimized the hardware design of the wireless microsystem, the current platform still relies on commercial off-the-shelf (COTS) components, which limits further reductions in size and weight. A natural next step is to develop a dedicated multimodal wireless acquisition integrated circuit (IC) and move toward an application-specific integrated circuit (ASIC)-based microsystem architecture. Second, the present microsystem supports only electrophysiological and electrochemical recording. Incorporating electrical or optogenetic stimulation to enable closed-loop operation [36,37], together with additional sensing modalities such as ion concentration or tissue oxygenation, would help establish a more comprehensive platform for neural monitoring and modulation. Finally, in the dexmedetomidine experiments, recordings were restricted to the mPFC, and upstream regions such as the VTA were not monitored simultaneously. Future studies using multi-region MEAs will be needed to track circuit-wide dynamics and clarify how activity along the VTA–mPFC pathway is reorganized under dexmedetomidine.

In conclusion, we developed a dual-mode wireless microsystem that supports simultaneous in vivo recording of spikes/LFPs and a DA-related amperometric response within the same cortical region. Using this platform, we observed concurrent changes across electrophysiological features and the DA-related electrochemical signal under graded dexmedetomidine dosing, demonstrating a compact approach for multimodal neural monitoring. These results establish a compact and practical platform for real-time multimodal neural monitoring and provide a foundation for future studies that integrate electrophysiological and neurochemical measurements to better characterize neural states, such as neural signal detection in disorder models, neurorehabilitation studies, and cognitive paradigms.

## Data Availability

All data needed to support the conclusions in the paper are provided in the paper and the Supplementary Materials. Representative raw data and additional datasets are available from the authors upon reasonable request. The analysis code used for signal processing is available from the authors upon reasonable request. Key system architecture details are provided in the paper and/or the Supplementary Materials, and further implementation details may be requested from the authors.

## Supplementary Materials

Supplementary 1 Figs. S1 to S6 Table S1 References [38,39]
