# Supplementary material for: A Dual-Mode Wireless Microsystem for Monitoring Dopamine and Spike Changes with Dexmedetomidine
Source: Cyborg Bionic Syst. 2026 May 21;7:0566. doi: 10.34133/cbsystems.0566 (PMC13191085; doi:10.34133/cbsystems.0566)
Supplement: Supplementary 1 — Figs. S1 to S6 Table S1 References [38,39] [file cbsystems.0566.f1.zip › figureS1.pdf]

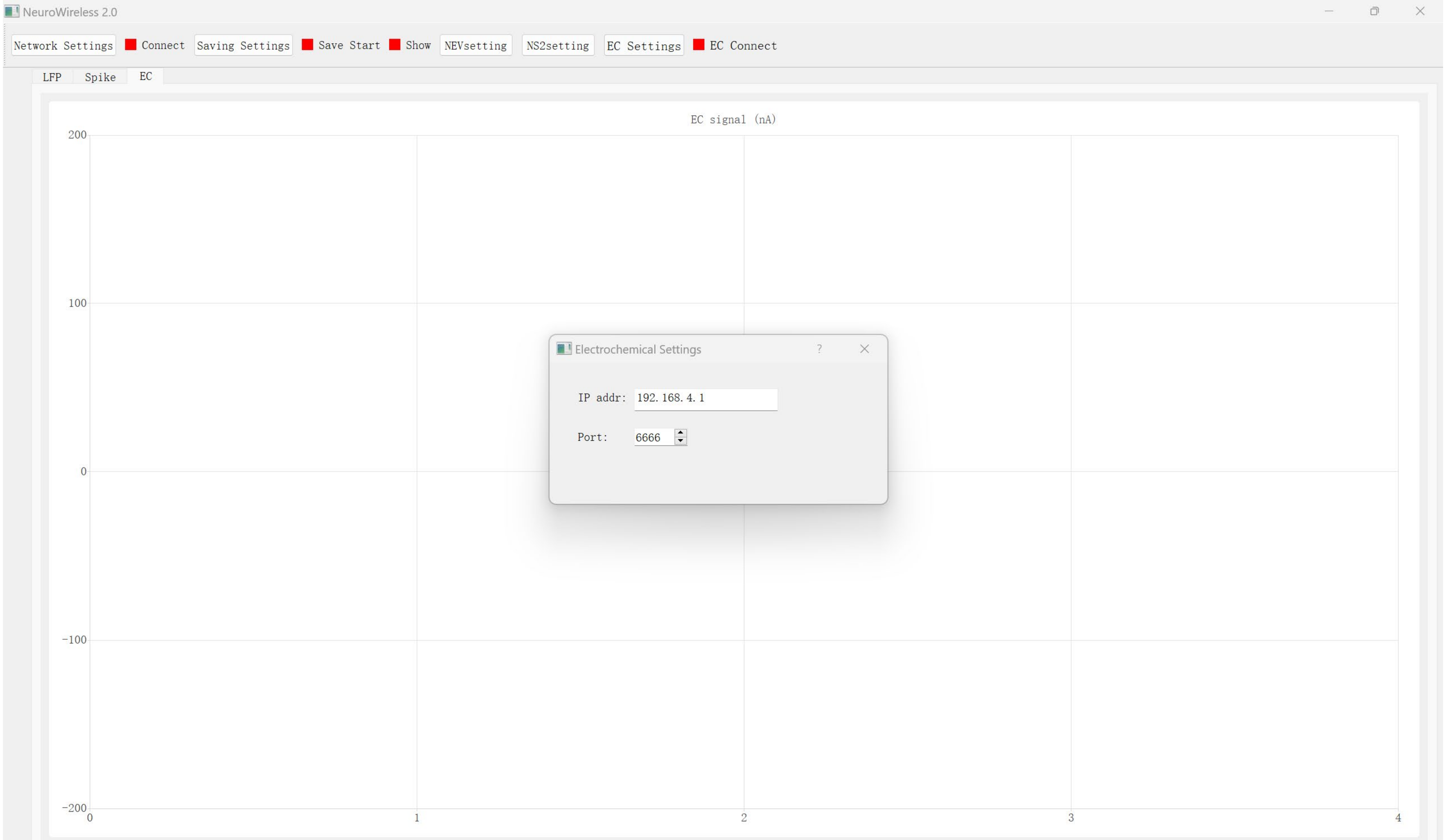
NeuroWireless 2.0
Network Settings
Connect
Saving Settings
Save Start
Show
NEVsetting
NS2setting
EC Settings
EC Connect
LFP
Spike
EC
EC signal (nA)
200
100
0
−100
−200
0
1
2
3
4
Electrochemical Settings
IP addr:
192. 168. 4. 1
Port:
6666
