# Supplementary material for: A Dual-Mode Wireless Microsystem for Monitoring Dopamine and Spike Changes with Dexmedetomidine
Source: Cyborg Bionic Syst. 2026 May 21;7:0566. doi: 10.34133/cbsystems.0566 (PMC13191085; doi:10.34133/cbsystems.0566)
Supplement: Supplementary 1 — Figs. S1 to S6 Table S1 References [38,39] [file cbsystems.0566.f1.zip › SUPPLEMENTARY MATERIALS from source.docx]

SUPPLEMENTARY MATERIALS

Figures S1 to S4


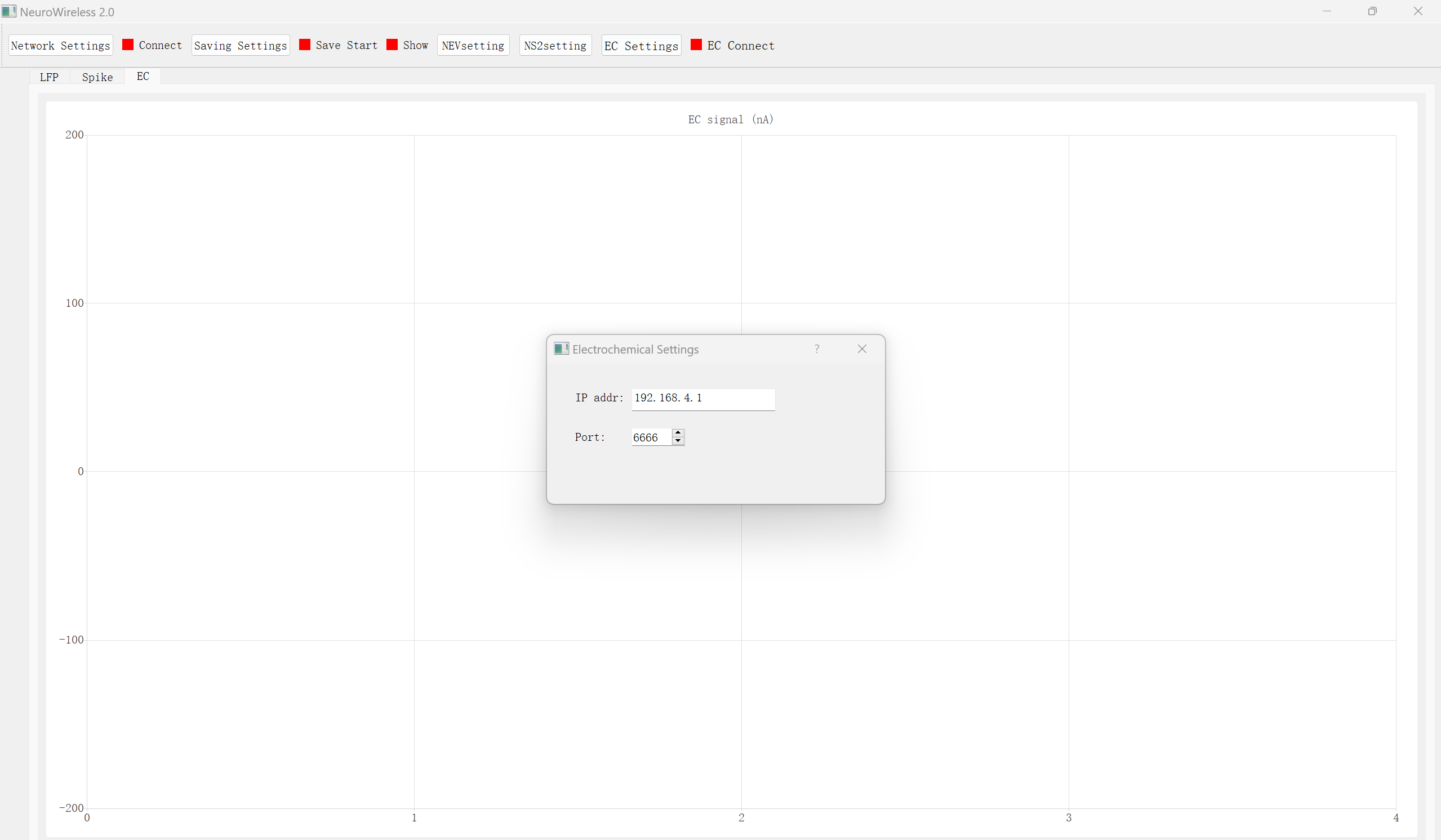


**Fig. S1. User interface of the upgraded NeuroWireless 2.0 software.** The software includes an added electrochemical (EC) module for wireless dopamine acquisition. Shown here is the EC settings dialog, where the IP address and port for EC data reception can be configured.

**Fig. S2. Scanning electron microscope (SEM) and energy dispersive spectrometer (EDS) analyses of microelectrode sites modified with different materials.** (A-C) Low-magnification SEM images of microelectrode sites modified with PtNPs, PtNPs/PEDOT:PSS, and PtNPs/PEDOT:PSS/rGO, respectively, showing overall surface morphology at the microelectrode scale. (D-F) High-magnification SEM images corresponding to panels A-C, illustrating nanoscale structural features of each modification. (G–I) Elemental composition obtained from EDS for the three modified microelectrodes, confirming the presence of Pt, C, and O associated with PtNPs and polymer layers, and the increased C and S content in the PtNPs/PEDOT:PSS/rGO coating. These results validate the successful formation of the composite rGO-enhanced sensing interface used for dopamine detection.

**Fig. S3. Cyclic voltammetry curves of the PtNP/PEDOT:PSS/rGO/Nafion-modified microelectrode measured in PBS and 200 μM dopamine at a scan rate of 100 mV/s (green and blue, respectively).**

**Fig. S4. Extended electrochemical characterization of the dopamine (DA)-sensing site.** (A) Amperometric responses to stepwise DA increments at 0.15 V at PtNPs/PEDOT:PSS/rGO/Nafion-modified microelectrode. (B) Expanded view of low-concentration responses, the area indicated by the dotted box in Fig. S4A, showing a detection limit of 10 nM. (C) Linear relationship between current and DA concentration. (D) Current responses to common interferents and comparison with DA.

**Fig. S5. Baseline drift test of the amperometric EC channel in PBS.** Amperometric current recorded continuously for 50 min in PBS to evaluate baseline stability. A slow baseline drift is observed over time. The fitted drift rate is 0.126 pA/min, and the detrended RMS is 2.60 pA.

**Fig. S6. Bidirectional cross-talk evaluation between EP and EC.** (A) Electrophysiology (EP) → electrochemistry (EC) test: PBS amperometric current recorded while EP streaming was enabled; the arrow marks the time when EP was enabled. A slight baseline shift is observed after EP enabling. (B) EC→EP test: EP signal recorded before and after enabling EC amperometric operation; the arrow marks the time when EC was enabled. No obvious increase in EP noise amplitude is observed after EC enabling.

**Table S1. Benchmarking against representative related works.**

| **Work** | **Liu**[15]  **(2020)** | **Bilodeau**[38]  **(2021)** | **Shin**[12]  **(2022)** | **Stuart**[39]  **(2023)** | **This work** |
| --- | --- | --- | --- | --- | --- |
| Modalities | EC (DA) | LFP/spike | LFP/spike | EC (DA) | **LFP/spike/EC (DA)** |
| Size (mm × mm) | 22×13 | 28×15 | 24×20 | 12×8.5 | **26×22** |
| Weight (g) | 2.0 | 1.7 | 2.44 | 0.049 | **8.99** |
| EP channels | N/A | 32 | 16 | N/A | **32** |
| EP sampling rate (kHz) | N/A | 20 | 8 | N/A | **30** |
| EC sensitivity  (as reported) | ~60 pA/μM | N/A | N/A | 86 mV/μM | **32.4 pA/μM (bench)**  **19.8 pA/μM (wireless)** |
| EC linearity (R²) | 0.997 | N/A | N/A | NR | **0.990 (bench)**  **0.988 (wireless)** |
| EC LOD (nM) | ~100 | N/A | N/A | ~300 | **10 (bench)**  **200 (wireless)** |
| Transmit distance | NR | NR | ~50 m | NR | **~20 m** |
| In vivo validation | VTA (mouse) | primary motor cortex (rat) | mPFC (mouse) | nucleus accumbens/striatum (mouse) | **PrL (rat)** |
| Stability (pA/min) | NR | N/A | N/A | ~11.7 | **0.126** |
| Time alignment | NR | NR | NR | NR | **mean offset 0.717 ms**  **jitter 1.42 ms** |
| Throughput (kB/s) | NR | 179.2 | <128 | NR | **370** |

EP, electrophysiology; EC, electrochemistry; DA, dopamine; LFP, local field potential; LOD, limit of detection; NR, not reported; N/A, not applicable. mPFC, medial prefrontal cortex; VTA, ventral tegmental area; PrL, prelimbic cortex. EC sensitivity is listed in the unit used in the original paper.
