# Supplementary figures and images for: A Dual-Mode Wireless Microsystem for Monitoring Dopamine and Spike Changes with Dexmedetomidine
Source: Cyborg Bionic Syst. 2026 May 21;7:0566. doi: 10.34133/cbsystems.0566 (PMC13191085; doi:10.34133/cbsystems.0566)

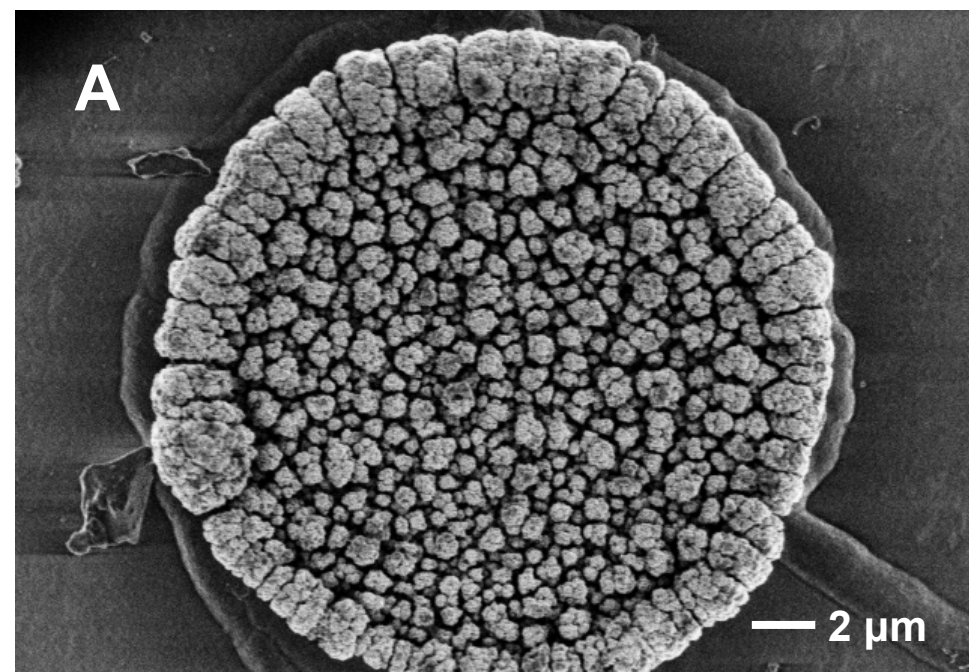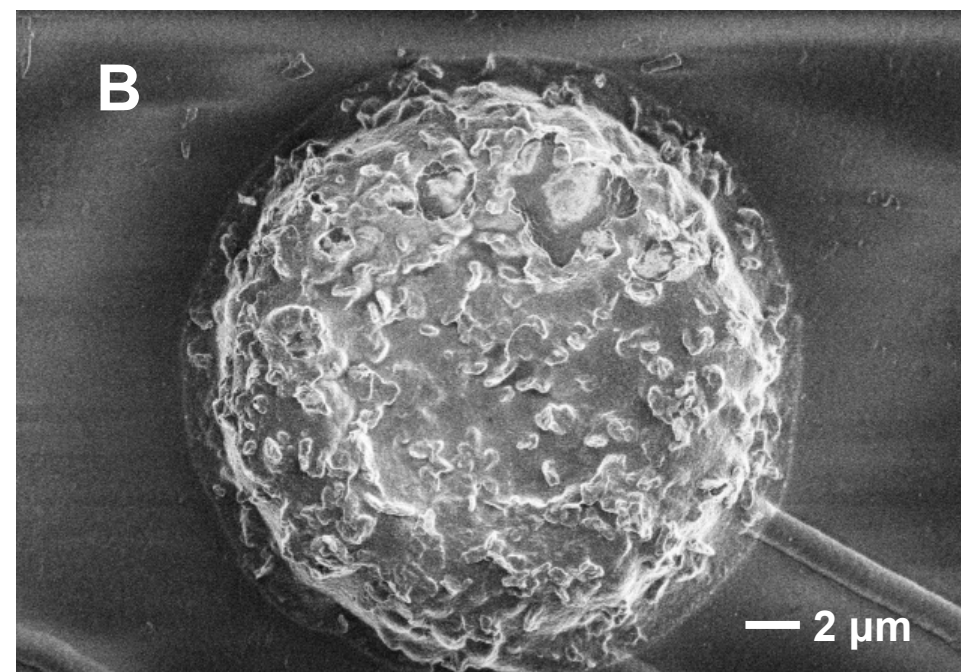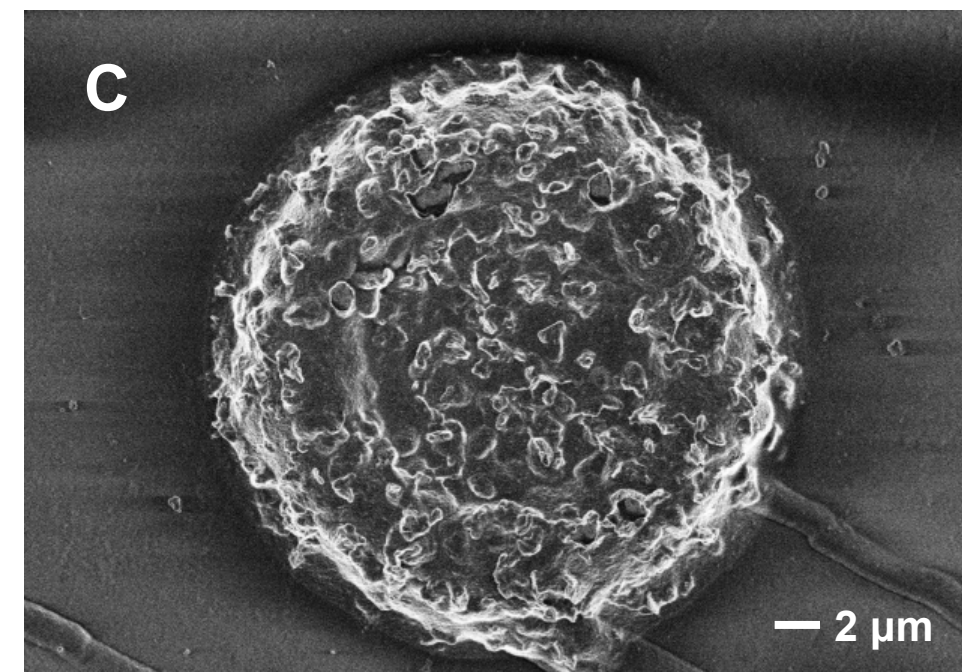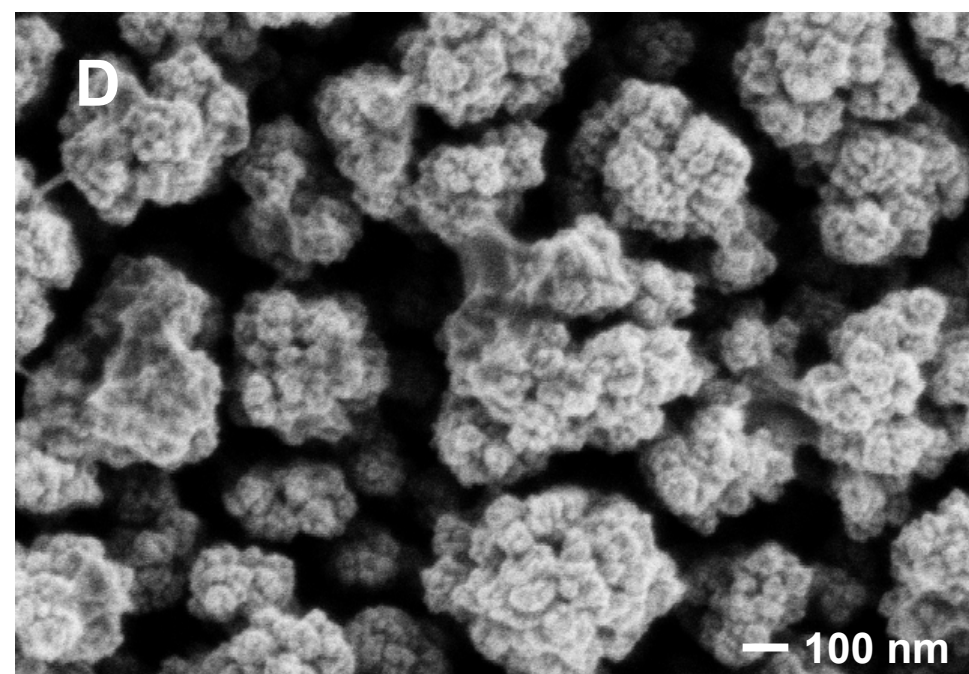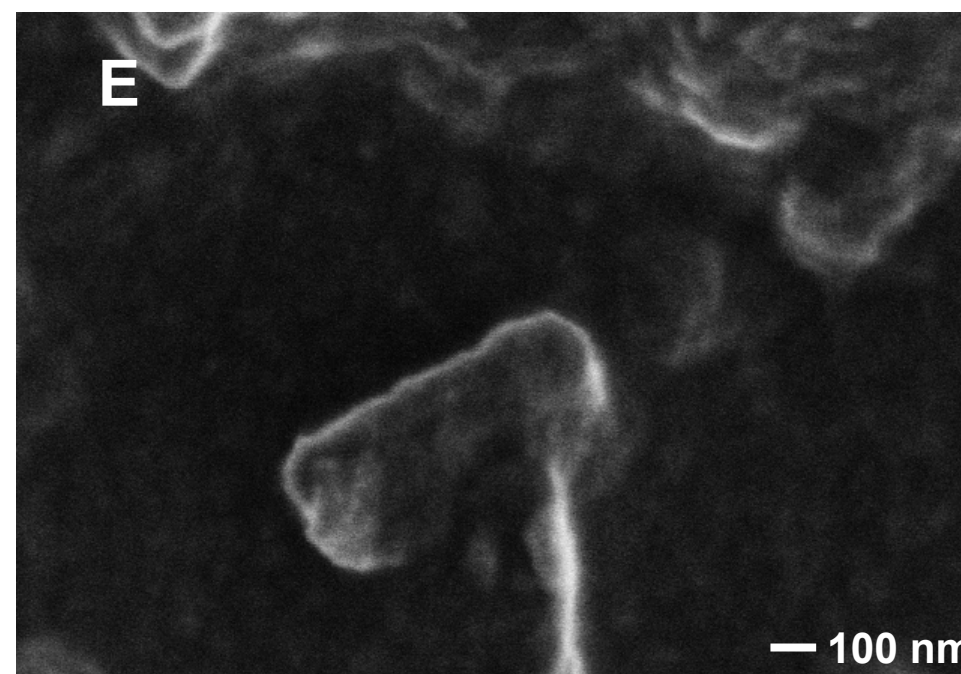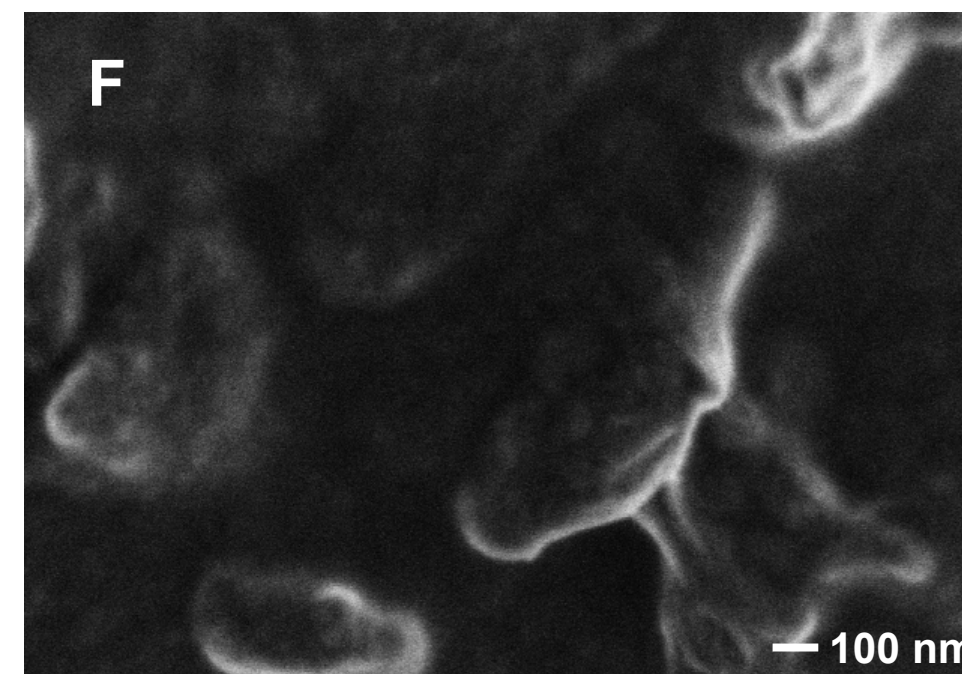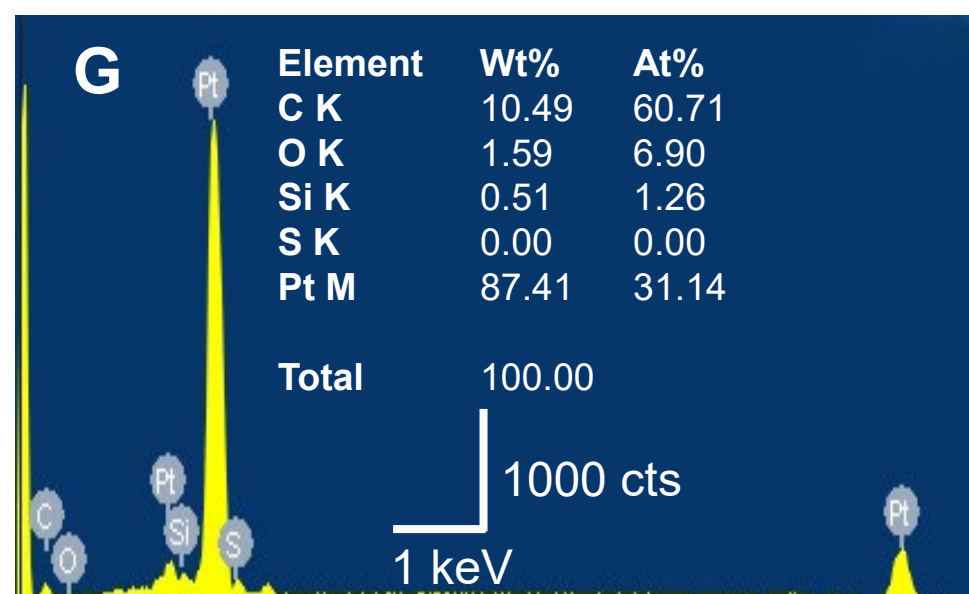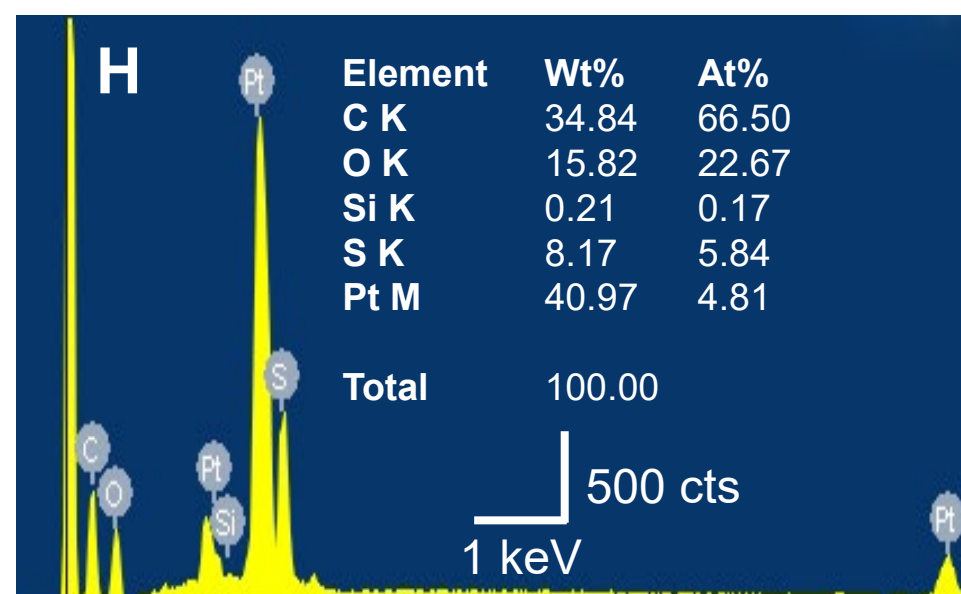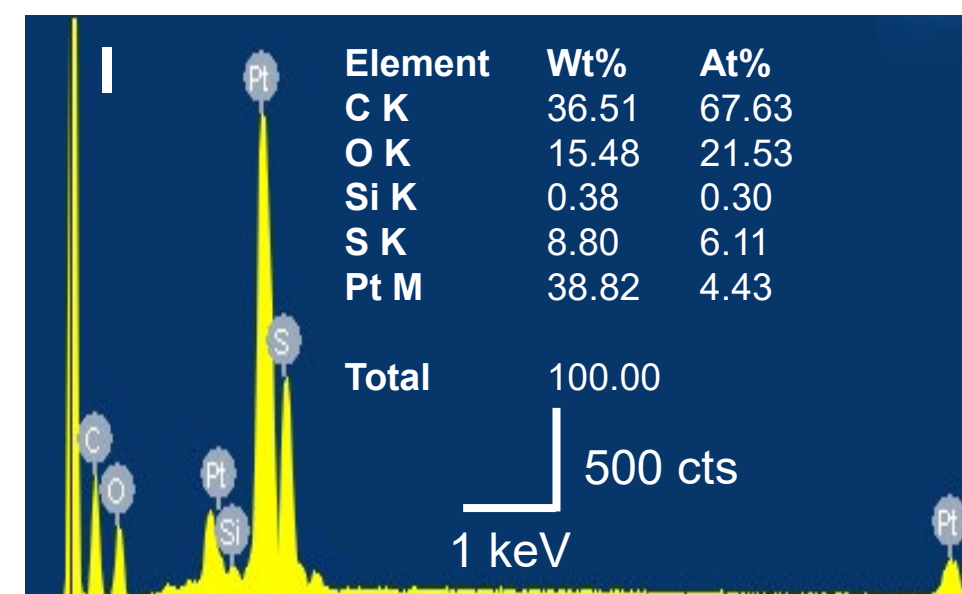

Supplement: Supplementary 1 — Figs. S1 to S6 Table S1 References [38,39] [file cbsystems.0566.f1.zip › figureS2.pdf]

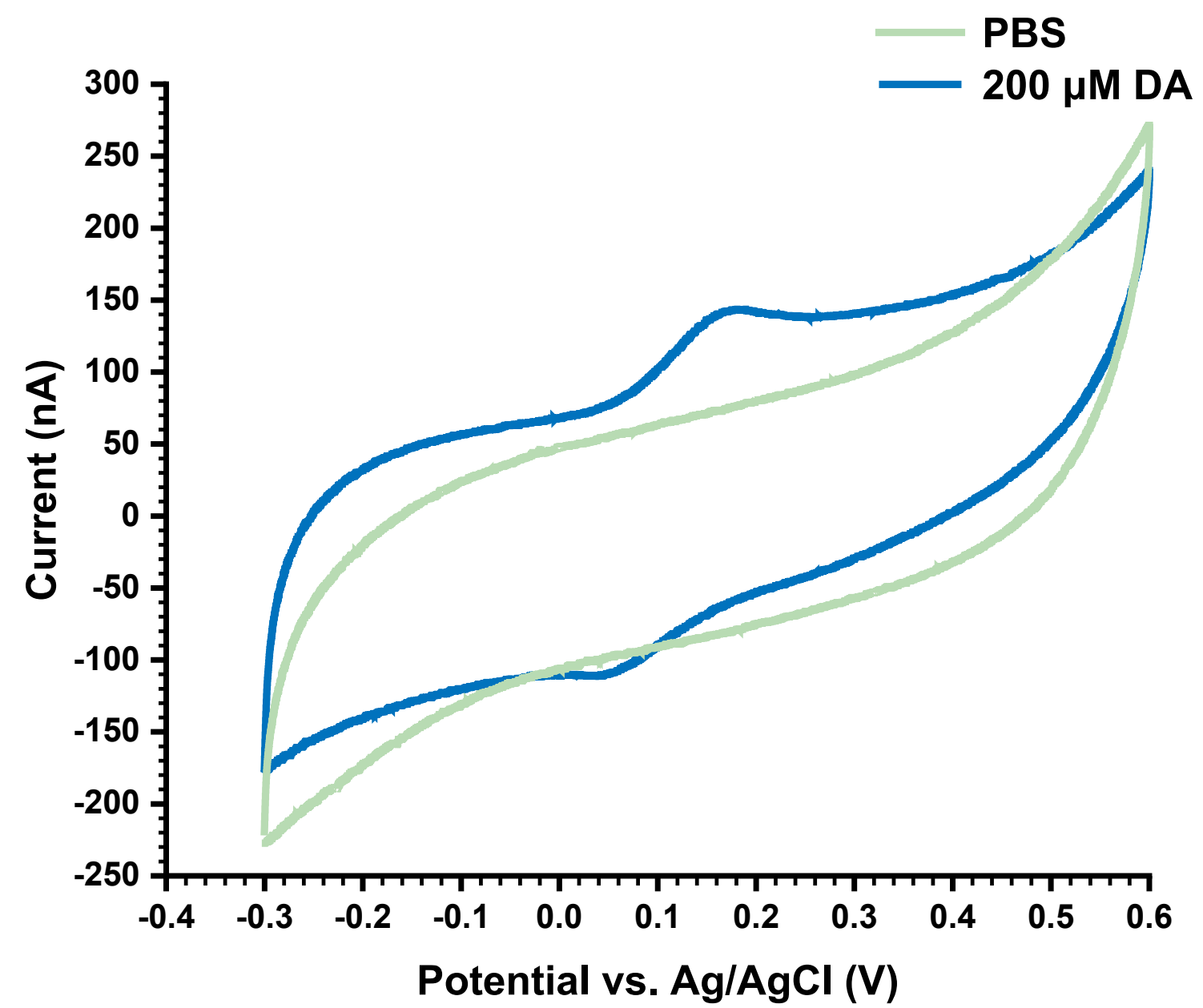

Supplement: Supplementary 1 — Figs. S1 to S6 Table S1 References [38,39] [file cbsystems.0566.f1.zip › figureS3.pdf]

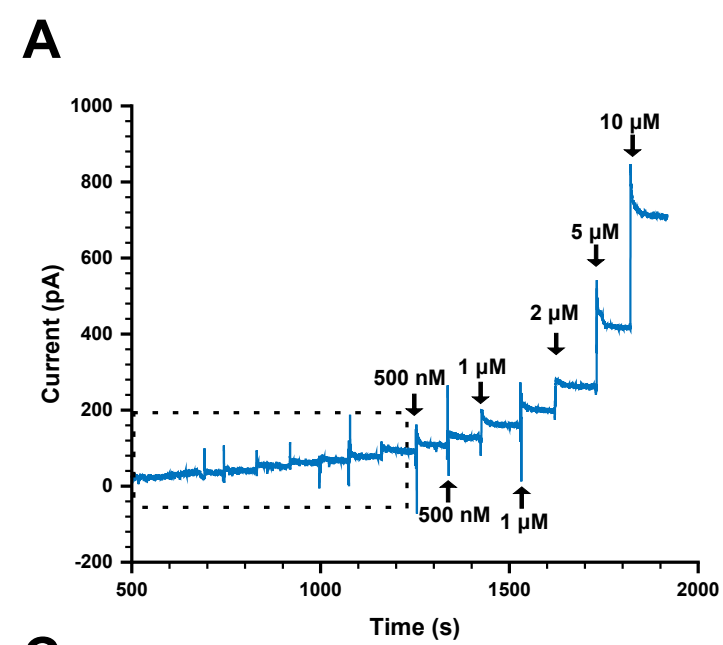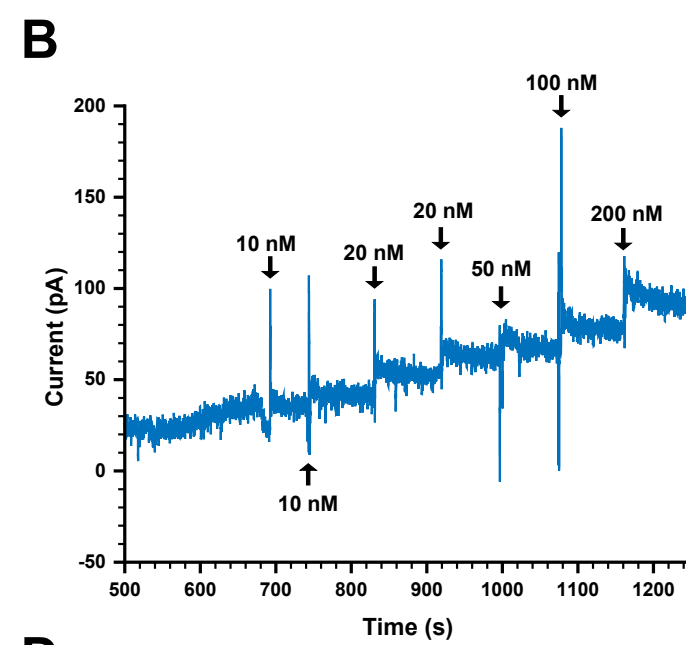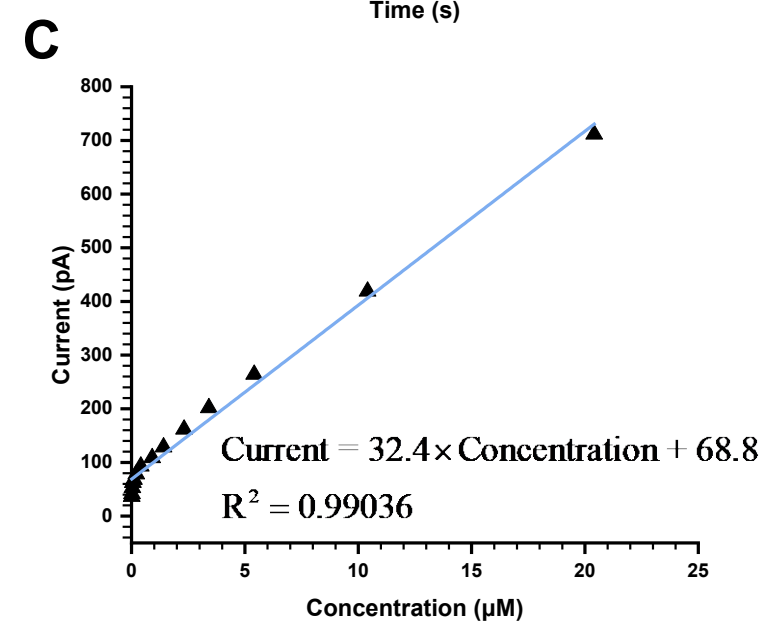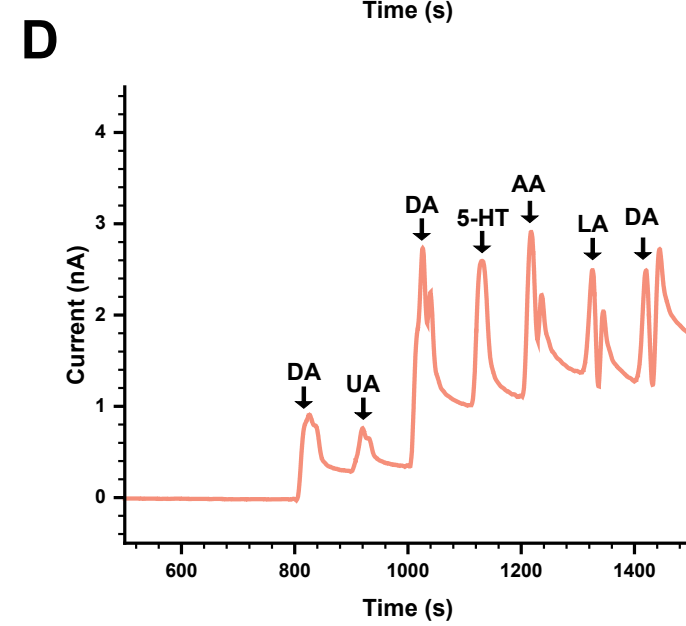

Supplement: Supplementary 1 — Figs. S1 to S6 Table S1 References [38,39] [file cbsystems.0566.f1.zip › figureS4.pdf]

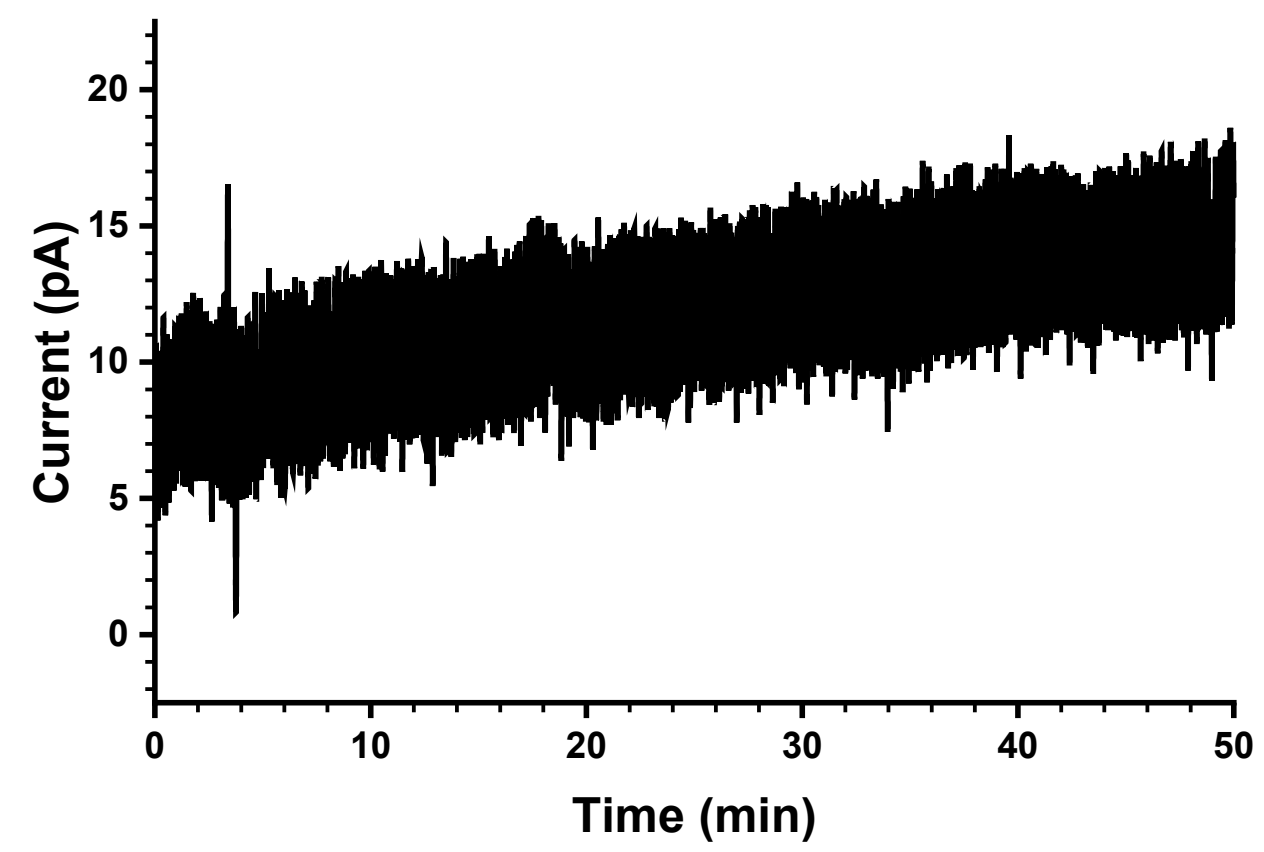

Supplement: Supplementary 1 — Figs. S1 to S6 Table S1 References [38,39] [file cbsystems.0566.f1.zip › figureS5.pdf]

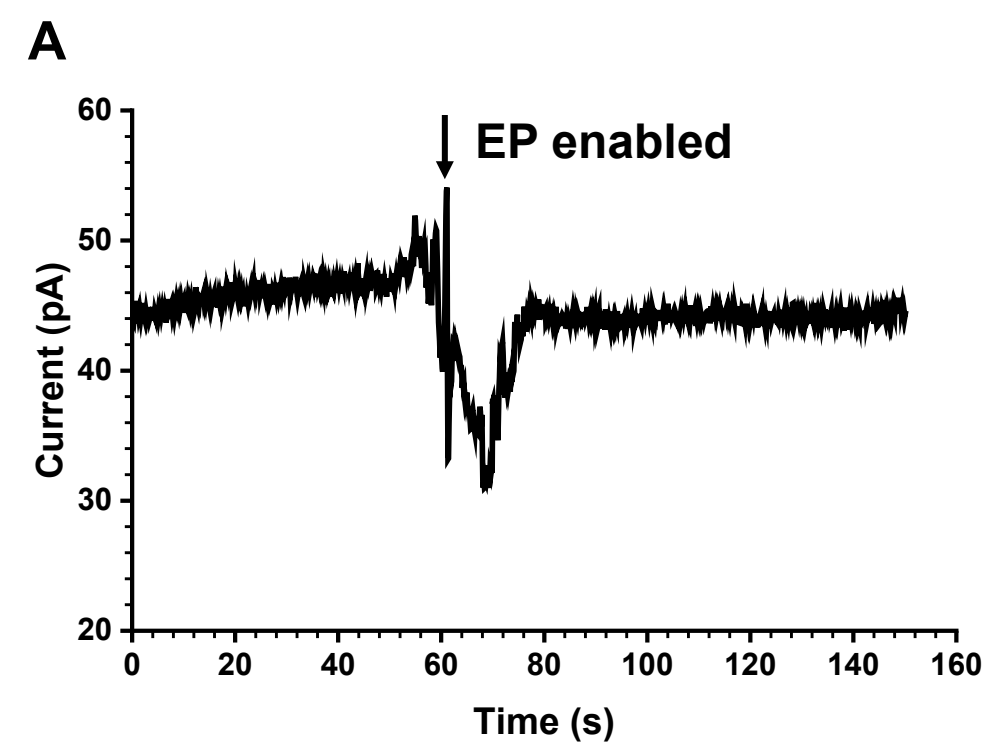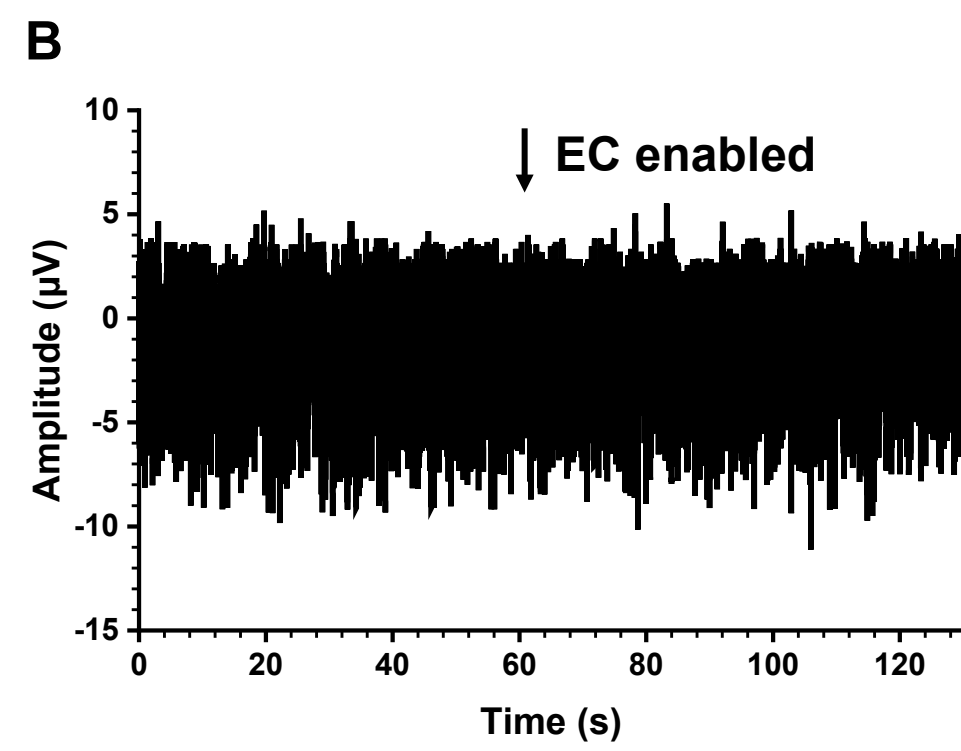

Supplement: Supplementary 1 — Figs. S1 to S6 Table S1 References [38,39] [file cbsystems.0566.f1.zip › figureS6.pdf]
